# Enhanced cell density cultivation and rapid expression-screening of recombinant *Pichia pastoris* clones in microscale

**DOI:** 10.1038/s41598-020-63995-5

**Published:** 2020-05-04

**Authors:** Neha Kaushik, Urpo Lamminmäki, Navin Khanna, Gaurav Batra

**Affiliations:** 1 Translational Health Science and Technology Institute, NCR Biotech Science Cluster, Faridabad, Haryana India; 2 School of Life Sciences, Manipal University, Manipal, 576104 Karnataka India; 3 Department of Biochemistry/Biotechnology, University of Turku, Turku, Finland; 4 Recombinant Gene Products Group, International Centre for Genetic Engineering and Biotechnology, New Delhi, India

**Keywords:** High-throughput screening, Microbiology techniques, Expression systems, Industrial microbiology, Applied microbiology

## Abstract

Cultivation of yeast *Pichia pastoris* in the microtiter plate, for optimisation of culture conditions, and expression screening of transformants has gained significance in recent years. However, in the microtiter plate, it has been challenging to attain cell densities similar to well-aerated shake-flask culture, due to the poor mixing resulting in oxygen limitation. To solve this problem, we investigated the influence of multiple cultivation parameters on *P. pastoris* cell growth, including the architecture of 96-deepwell plate (96-DWP), shaking throw diameter, shaking frequency, culture volume/well, and media composition. In the optimised conditions, a cell density of OD_600_ ~50 (dry cell weight ~13 g/L) with >99% cell viability was achieved in the casamino acids supplemented buffered-minimal-media in 300 to 1000 *μ*l culture volume/well. We have devised a simplified method for coating of the culture supernatant on the polystyrene surface for immunoassay. Clones for secretory expression of envelope domain III of dengue virus serotype-1 under the control of inducible and constitutive promoter were screened using the developed method. Described microscale cultivation strategy can be used for rapid high-throughput screening of *P. pastoris* clones, media optimization, and high-throughput recombinant protein production. The knowledge gained through this work may also be applied, to other suspension cultures, with some modifications.

## Introduction

In the past two decades, yeast *Pichia (Komagataella) pastoris* has become one of the most popular expression systems for the production of recombinant proteins of commercial utility. Being a lower eukaryote, it possesses dual attributes of both prokaryote (easy and economical handling, high cell density) and eukaryote (equipped for performing many post-translational modifications)^1^. *P. pastoris* secretes very low levels of endogenous proteins, and the presence of low amount of host proteins in *P. pastoris* culture supernatant simplifies the downstream processing for secretory recombinant proteins^2–4^.

For the generation of stable clones, genomic integration of expression cassette is preferred in *P. pastoris*. The expression vector is targeted to integrate at the desired locus of the host genome via homologous recombination. In this manner, recombinant strains containing single or multiple copies of the expression cassette are generated. This process results in clonal heterogeneity because of the differences in the number of expression cassette copies integrated and at times off-target integration. Therefore, screening of a large number of transformants is required to distinguish the clone with the highest level of expression and secretion^4^.

The optimal expression conditions may vary for different recombinant proteins particularly for the secreted proteins, as the media composition may affect the stability of the secreted recombinant product^3^. Thus, high throughput screening (HTS) of expression conditions is often required to identify the most optimal conditions^5^. In recent years, high throughput parallel production of recombinant proteins in microwells has also gained popularity, particularly for combinatorial biology applications requiring an efficient microscale cultivation process for handling a large number of different variants^6^. Microscale cultivation of yeast is also necessary for the screening of co-expression library of secretion factors for efficient secretion of recombinant protein, e.g., antibody^7^.

There are several reports of cultivation and expression of recombinant proteins in *P. pastoris* in deepwell plates (DWP) to identify the suitable clone or condition. Boettner *et al*. described a screening system for high-throughput characterization of *P. pastoris* expression clones in 2 ml culture volume in a 24-well plate format^8^. In another study, 48-well plate was applied for the screening of engineered constitutive promoters using yeast-enhanced green fluorescent protein as a reporter in *P. pastoris*^9^. In yet another studies, 96-DWP was utilized with 500 *μ*l to 600 *μ*l culture volumes for expression screening of enzyme variant and monoclonal antibodies^10,11^. However, only two studies reported the cell density of *P. pastoris* in terms of optical density (OD_600_ 10–12)^10^ or dry cell weight (DCW, 3.3 g/L)^9^ attained in DWP. The reported values indicate remarkably lower growth in DWPs in comparison to that achievable in well-aerated shake flask^3^.

The vast difference, in the cell densities in DWP and the production scale, does not allow reliable prediction of appropriate clone or conditions in DWP. Moreover, the low-density low-volume culture conditions cannot be used for efficient high throughput protein production in DWP. Nevertheless, an in-depth analysis of the conditions that promote adequate mixing and higher cell growth in microtiter plate has not been reported for *P. pastoris*.

The major limitation of microbial cultivation at microscale is the inadequate oxygen transfer rate due to poor mixing^12,13^. Although parameters such as dissolved oxygen level, pH and feed rates, are routinely controlled and monitored via the online measurement system in the bioreactor, these are often not taken into account with routine DWP culture. In recent years, microtiter plates, with non-invasive optical monitoring system, called microbioreactors, have also been developed^13,14^. However, these are not feasible for large-scale screening. Moreover, the microbioreactor are often not available in simple laboratory settings. In this study, we have optimized multiple cultivation parameters for *P. pastoris* in 96-DWP achieving cell density (OD_600_ ~50; dry cell weight ~13 g/L) and recombinant protein expression similar to the well-aerated shake flask culture. We confirmed that the optimized conditions provide uniform cell growth and expression for an identical clone in all wells of a DWP. The method established by us is well suited for automation and applicable for parallel expression screening of a large number of clones or cultivation conditions under the inducible as well as the constitutive promoter in *P. pastoris*. We have used dengue virus (DENV) envelope domain-III (EDIII) as model protein in this study. The identity amongst the EDIIIs of the four DENV serotypes is 60–70%^3^, and these antigenically distinct domains can be used for the serotyping of immune response^15^.

## Methods

### Microbial host strain and vectors

*E. coli* DH5α and *P. pastoris* Mut^s^ strain KM71H were procured from Thermo Fisher Scientific Corporation. The *P. pastoris* integrative plasmids pD912 and pD915 were obtained from Atom Inc. (previously DNA 2.0). Both the vectors contain *Saccharomyces cerevisiae* derived α-prepro signal sequence, alcohol oxidase 1 (AOX1) terminator, zeocin resistance marker and pUC ori sequence for propagation in *E. coli*. Vector pD912 has methanol inducible AOX1 promoter, while constitutive glyceraldehye-3-phosphate dehydrogenase (GAP) promoter is present in pD915.

### Other reagents

All the molecular biology enzymes, *P. pastoris* codon optimized EDIII gene of dengue virus serotype-1 (DENV-1) with 6×-His tag at 3′end, zeocin antibiotic, LIVE/DEAD FungaLight yeast viability kit, 96-DWP (square wells with V-shaped bottom; 2.2 ml total volume), breathable rayon tape, anti-His mAb and UltraPure DNase/RNase-free distilled water were purchased from Thermo Fisher Scientific Corporation, USA. 96-DWP (square wells with U-shaped bottom; 2.2 ml total volume per well) was from Genetix Biotech Asia Pvt Ltd, India. Goat anti-mouse IgG-H&L-chain was procured from Jackson ImmunoResearch Laboratories, Inc. USA. N1-europium chelate was synthesized at University of Turku, Finland. YeaStar genomic DNA isolation kit was purchased from Zymo Research, CA, USA. 2x SSO EvoGreen mix, hard-shell white 96-well PCR plate with clear wells and microseal ‘B’ adhesive sealing film were obtained from Bio-Rad Laboratories, CA, USA. All the other chemicals were procured from Sigma-Aldrich Corporation, USA and culture media and casamino acids were purchased from Becton, Dickinson and Company, USA. The casamino acids (CA) is acid hydrolysed casein with low sodium chloride and iron concentrations (Bacto^TM^ casamino acids Cat # 223050). Peptone is an enzymatic digest of animal protein (Bacto^TM^ peptone Cat # 211677). Primers for quantitative PCR (qPCR), were synthesized by IDT, Singapore.

### Optimization of 96-DWP based cultivation conditions

A pre-culture was set up by inoculating 50 ml of YPD (1% Yeast extract, 2% Peptone, 2% Dextrose) medium with a glycerol stock of pre-existing *P. pastoris* secretory clone of dengue virus serotype-3 (DENV-3) EDIII in shake flask and grown till optical density (OD_600_) of 2. An aliquot from primary culture was inoculated into growth medium to a starting OD_600_ of 0.1 and dispensed in different volumes (300 *μ*l to 1000 *μ*l) per well of sterile 96-square DWP. Culture-filled wells were spaced by well containing 600 *μ*l of sterile media. Plates were sealed with sterile breathable rayon tape and incubated at 30 °C on orbital shakers (climo-shaker ISF1-X, Kühner AG, Switzerland) of different throw diameter. Culture aliquots were withdrawn after 72 h, and the cell density and viability were determined. For the optimization, we tested three different growth media: YPD, buffered minimal glycerol (BMG) [100 mM Potassium phosphate, pH 5.8; 1.34% Yeast Nitrogen Base; 0.4 μg/ml Biotin; 1% Glycerol] and BMG supplemented with CA [BMG-CA (CA = 0.5% w/v)]. In the initial set of experiments, the impact of bottom geometry of 96-DWP with square wells (U-shaped vs. V-shaped bottom) on *P. pastoris* cell growth was evaluated using YPD medium. Both types of plates were incubated at 25 mm throw diameter shaker with shaking frequency of 300 rpm and 400 rpm and 50 mm throw diameter shaker with a shaking frequency of 300 rpm. Next, 3 mm throw diameter shaker with various shaking frequencies such as 500, 650, and 850 rpm was tested using U-bottom 96-DWP. Subsequently, 1000 rpm shaking frequency of 3 mm throw diameter shaker was tested using V- and U-bottom 96-DWP. Later, the effect of minimal media (BMG and BMG-CA) on cell density [in terms of OD_600_ & dry cell weight (DCW)] and cell viability of *P. pastoris* in U-bottom plate was assessed with shaking at 1000 rpm in 3 mm throw diameter shaker.

### Optimization of 96-DWP based expression conditions

For determination of culture duration, different size colonies of *P. pastoris* KM71H were grown in 96-DWP containing 600 µl of YPD media per well. The cultures were grown under the optimized cultivation conditions for 96 h. Culture aliquots were withdrawn at every 24 h, and OD_600_ was measured.

In another experiment, a starter culture was set up by inoculating 50 ml of YPD with a glycerol stock of *P. pastoris* DENV-3 EDIII clone in a flask. This was grown at 30 °C, 250 rpm, for 24 h and used to inoculate 130 ml of BMG-CA media to a starting OD_600_ 0.1 and distributed into a sterile 96-square DWP (U-bottom) with 600 *μ*l culture volume per well. Plates were shaken with 1000 rpm (3 mm throw diameter shaker) at 30 °C for 48 h. Cells were pelleted at 4000 × g, 5 min and re-suspended in 550 *μ*l of corresponding induction media, buffered minimal methanol supplemented with CA (BMM-CA), where glycerol was replaced by 2% methanol. Induction was allowed to proceed for 48 h at 20 °C. Cultures were centrifuged, and clarified supernatants were further used for the determination of secreted EDIII amount by immunoassay.

### Generation of DENV-1 EDIII expressing *P. pastoris* clones

*P. pastoris* optimized, synthetic gene encoding DENV-1 EDIII was cloned under the control of AOX1 promoter with α-pre-pro signal peptide in pD912 vector, using SapI restriction enzyme-based cloning. The resultant ligated product was transformed into *E. coli* DH5α, and the isolated plasmid was linearized at a unique SacI restriction site in the AOX1 promoter (PAOX1). Similarly, DENV-1 EDIII gene was cloned into the pD915 vector containing GAP promoter followed by α-pre-pro signal peptide, and the resultant plasmid was linearized at unique AvrII restriction site within the GAP promoter (PGAP). Both linearized plasmids were purified with QIAprep spin column, sequence verified and electroporated into *P. pastoris* KM71H according to the condensed cell protocol^16^ to generate PAOX1 and PGAP based clones. Transformants were selected on YPDS agar (1% Yeast; 2% Peptone; 2% Dextrose; 1% Sorbitol; 2% Agar) plates containing 200 µg/ml of zeocin and incubated at 30 °C for 3 days.

### Screening of *P. pastoris* clones expressing DENV-1 EDIII

Sterile 96-DWP (U-bottom) filled with 600 *μ*l YPD per well was inoculated with individual colonies (50 clones) of *P. pastoris* transformed with DENV-1 EDIII (PAOX1 constructs). Additionally, glycerol stock of *P. pastoris* KM71H containing empty vector pPICZαA as a negative control was also inoculated in duplicate wells. The plate was incubated at 30 °C, 3 mm throw diameter shaker with 1000 rpm, for 48 h. From this pre-culture, 50 *μ*l aliquot was inoculated to a fresh sterile plate containing 600 *μ*l of BMG-CA media per well and incubated at 30 °C for 48 h. At the same time, an equal volume of 50% glycerol (final 25% v/v) was added to the remaining volume of the primary culture plate, sealed and stored at −80 °C as glycerol stock. After 48 h of growth, cells were pelleted at 4000 × g, 5 min and re-suspended in 550 *μ*l of BMM-CA for induction of AOX1 promoter and incubated for next 48 h at 20 °C. After induction, clarified supernatants were used for SDS-PAGE analysis and immunoassay for the determination of EDIII secretion level. Similarly, screening of constitutive GAP promoter based *P. pastoris* clones of DENV-1 EDIII (50 colonies) was performed, except that they were grown only in BMG-CA media for 48 h and no separate induction step was performed.

### Copy number determination of *P. pastoris* strain expressing DENV-1 EDIII

Genomic DNA of *P. pastoris* KM71H strain (untransformed) and *P. pastoris* KM71H transformed with DENV-1 EDIII (PAOX1 based construct) was isolated according to the protocol described in YeaStar Genomic DNA Kit. Two clones of *P. pastoris* expressing DENV-1 EDIII under the control of PAOX1, viz., clone number 5 and clone number 39, being low and high expressor, respectively, were selected for the determination of copy number of EDIII inserted in the genome of *P. pastoris*. For this purpose, primers were designed using the PrimerQuest qPCR assay design tool (IDT, Coralville, IA). PAOX1 region of the expression cassette was targeted for copy number determination of DENV-1 EDIII containing *P. pastoris* strains and sequence of primers were as follow: forward primer 5′-GAAGCTGCCCTGTCTTAAACCTT-3′ and reverse primer 5′-CAAAAGCTTGTCAATTGGAACCA-3′. GAPDH gene was used as an endogenous control as this is a single copy gene of the haploid host and primer sequence for GAPDH gene was as follows: forward primer 5′-ATGACCGCCACTCAAAAGACC-3′ and reverse primer 5′-TTAGCAGCACCAGTGGAAGATG-3′. All qPCR amplification was performed using Bio-Rad CFX96 Touch real-time PCR cycler instrument with CFX manager detection software. qPCR mixtures were prepared using 2× SSO EvoGreen mix. For single qPCR reaction, 500 nM of each primer and 0.2 ng genomic DNA were added to 1× EvoGreen mix in reaction volume of 20 *μ*l. For each unknown (or test) *P. pastoris* strain (low and high expressor), reactions with PAOX1 and GAPDH primer sets were performed in separate well. Reactions were performed in triplicate with a standard curve and non-template control (NTC) for each gene recorded in every plate (Bio-Rad hard-shell white 96-well PCR plate, low profile, semi-skirted with clear wells). The thermal profile initiates with a 3 min step at 95 °C followed by 40 cycles of 30 sec at 95 °C and 30 sec at 55 °C. The amplification period was followed by a melting curve analysis with a temperature gradient of 0.5 °C per 0.005 sec from 55 °C to 95 °C to exclude amplification of non-specific products. The standard curve for each gene covered a copy quantity range from 2.0 × 10^6^ to 2.0 × 10^2^ copies per reaction. Calculation of copy number was based on the genome size of 9.43 Mbp^17^ resulting in approximately 94,300 copies of the genome present in 1 ng haploid *P. pastoris* genomic DNA. The mean Cq values were plotted against the log10 of their initial template copy quantity and standard curves were generated by linear regression of the plotted points. For copy number determination, absolute and relative quantification calculation was performed as described before^18^. The endogenous PAOX1 copy has been subtracted from the total PAOX1 copies determined.

### Expression in shake flask

The clone #39, one of the best secretor, harbouring 8 expression cassettes and a low secretor clone #5, harbouring 1 expression cassette were checked for expression in shake flask. Cultivation and expression protocol of shake-flask culture was followed as described earlier^3^, with notable changes like single concentration of CA (0.5% w/v) was added to media (BMG and BMM) and the induction was maintained only for 48 h at 20 °C without methanol pulsing. In parallel, these two clones (#5 and #39) of DENV-1 EDIII were also inoculated in 96-DWP (U-bottom) for comparison purpose, and expression procedure was followed as described earlier in the method section. After 48 h of induction, clarified supernatants were used for SDS PAGE and immunoassay for the determination of EDIII secretion.

### Analytical methods

The cell density was determined by measuring OD at 600 nm (OD_600_) of the appropriately diluted culture. For dry cell weight (DCW) determination, culture aliquots were centrifuged in pre-weighed microcentrifuge tubes, and the pellet was dried at 80 °C hot air bath as described earlier^19^. Cell viability was estimated by propidium iodide and syto9 staining using flow cytometer according to the manufacturer’s protocol of LIVE/DEAD FungaLight Yeast Viability Kit. For sample preparation, replicates from different wells of 96-DWP were used.

The interference of induction media components on binding of DENV-3 EDIII on polystyrene (maxisorp, thermo scientific) plate surface was evaluated using customized immunoassay. The purified DENV-3 EDIII protein was diluted in different induction media [BMM-CA, BMM and BMMY (1% Yeast Extract, 2% Peptone, 100 mM potassium phosphate buffer pH 5.8, Yeast Nitrogen Base; 0.4 μg/ml Biotin; 2% methanol)] and 1× PBS, pH 7.4 (50 mM Phosphate buffer, pH 7.4, 150 mM NaCl) to 10 *μ*g/ml. The media diluted protein was further diluted 1:100 in 50 mM carb-bicarb buffer (pH 9.6) and 100 µl of this was coated on maxisorp polystyrene plate wells (effective amount 10 ng EDIII protein per well) and incubated at 4 °C for overnight. The wells were washed 2 times with 1× PBS, pH 7.2, and incubated for 2 h in blocking buffer (1% BSA in 1× PBS, pH 7.4). After washing the blocked wells with wash buffer (5 mM Tris-HCl, pH 7.75, 150 mM NaCl, 0.02% sodium azide, 0.005% Tween-20) 3 times, 100 µl of anti-6×His-tag MAb as primary antibody (100 ng/ml) diluted in assay buffer (50 mM Tris-HCl pH 7.75, 150 mM NaCl, 0.01% Tween-40, 0.05% sodium azide, 20 *μ*M diethylenetriaminepentaacetic acid, 0.5% BSA, and 0.002% amaranth) was added and incubated for 1 h at 37 °C. The wells were washed 4 times with wash buffer and incubated with 100 µl of europium labeled secondary goat anti-mouse IgG (H + L) (1 µg/ml diluted in assay buffer), for 1 h at 37 °C. The plates were washed again, 4 times and the europium enhancement solution was added for signal generation. The plates were incubated for 15 min at 25 °C with gentle shaking. The europium counts were measured using PerkinElmer Envision multimode reader (excitation: 340 nm, and emission: 615 nm).

The immunoassay related to clone screening and EDIII level determination were also performed as explained above, except the culture supernatants were diluted 1:100 in 50 mM carb-bicarb buffer (pH 9.6). Each sample was assayed in duplicate. In parallel, a reference curve was generated using serially diluted (2-fold) purified DENV-1 EDIII protein of known concentration, and R^2^ value was 0.99.

## Results

### Impact of plate geometry, culture volume, media composition and shaking parameters on the growth and viability of *P. pastoris* in 96-DWP

**Figure 1:**
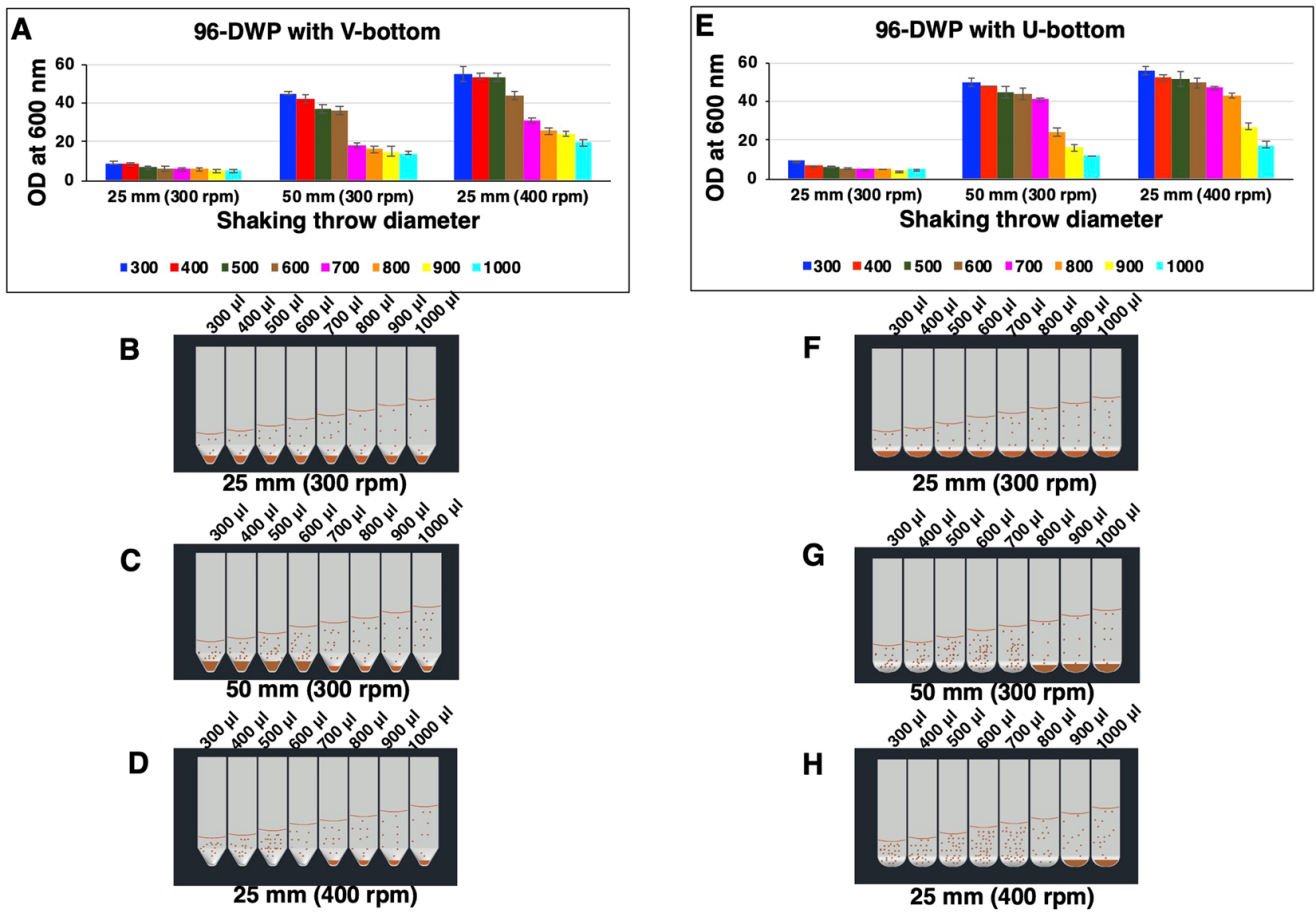
Impact of shaking throw diameter, plate geometry and culture volume on the cell growth. Parallel cultures, in YPD media, were grown in V-bottom (Panel A-D) and U-bottom (Panel E-H) square 96-DWP. Various culture volumes, ranging from 300 μl to 1000 µl per well, were used. Both the plates were incubated at 300 rpm on 25 mm and 50 mm throw diameter shaker as well as at 400 rpm on 25 mm throw diameter shaker. Culture aliquots were withdrawn after 72 h, and optical density was measured at 600 nm. In panel A and E, OD_600_ of the cultures are shown on the y-axis. Different colour bars represent different culture volume per well. Each data point represents the average of the 3 independent experiments. Bottom panels B, C, D, F, G and H show representative of visual observation of the settlement of cells.

In the first series of experiments, we examined the impact of the geometry of 96-DWP and other conditions i.e., shaking throw diameter, shaking frequency and culture volume per well on *P. pastoris* cell growth. This experiment was performed using a pre-existing *P. pastoris* secretory clone of dengue virus serotype-3 (DENV-3) EDIII^3^. We tested 2 types of square 96-DWP (V- and U-shaped bottom), filled with culture volumes from 300 *μ*l to 1000 *μ*l per well. After 72 h of incubation, cells settlement, culture density, and dry cell weight were determined. Figure S1 shows the schematic representation of the well geometry of 96-DWP. On 25 mm throw diameter shaker with 300 rpm, poor growth (OD_600_ 5 to 9) was attained in both V- and U-shaped bottom DWP with cells settlement with all the culture volumes tested (Fig. 1A,E). In striking contrast, when shaking was performed on 50 mm throw diameter shaker, biomass level showed a remarkable enhancement mainly in lower culture volumes tested (OD_600_ 36 to 45 in 600 to 300 µl in V-bottom and OD_600_ 41 to 50 in 700 to 300 µl in U-bottom 96-DWP) (Fig. 1A,E). However, cells settlement was observed in all culture volumes of V-bottom wells (Fig. 1C), whereas, in the case of U-bottom wells, cells settlement was only observed at higher culture volumes, i.e. 800 *μ*l to 1000 *μ*l (Fig. 1G). Next, when shaking frequency of 25 mm throw diameter shaker was increased from 300 rpm to 400 rpm, further enhancement in cell growth was observed (OD_600_ 19.5 to 55 in 1000 *μ*l to 300 *μ*l in V-bottom and OD_600_ 17.5 to 56 in 1000 *μ*l to 300 *μ*l in U-bottom 96-DWP) (Fig. 1A,E). However, cell settlement was observed in 700 *μ*l to 1000 *μ*l culture volume of V-bottom wells (Fig. 1D), whereas, in case of U-bottom wells, cell settlement was observed only in 900 *μ*l to 1000 *μ*l (Fig. 1H). This comparison of plate’s geometry shows that the U-bottom of square 96-DWP is better than V-bottom to maintain the cells in suspension at least for higher volumes.

**Figure 2:**
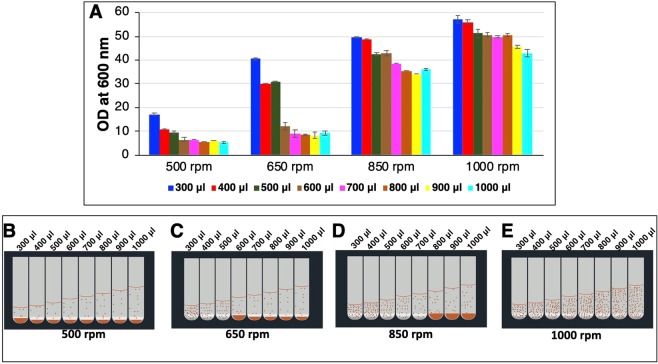
Effect of shaking frequency on the cell growth in 96-DWP on 3 mm throw diameter shaker. Cells were grown in different culture volumes (300 µl to 1000 µl per well) in YPD media in square 96-DWP with U-bottom. Aliquots were withdrawn after 72 h of cultivation and OD were measured at 600 nm. OD_600_ of the cultures is plotted on the y-axis in panel A. Different colour bars represent different culture volume per well. Each data point represents the average of 2 independent experiments. Panel B, C, D, E show representative of visual observation of the settlement of cells.

Next, we tested shaking at 3 mm throw diameter with shaking frequencies of 500, 650 and 850 rpm for U-bottom plate. At 500 rpm, cells settlement was observed in all the culture volumes (1000 to 300 *μ*l) (Fig. 2B) with poor growth (OD_600_ 5 to 17) (Fig. 2A). Successively, at 650 rpm, there was an improvement in the cell density (OD_600_ 31 to 41) (Fig. 2A) without cells settlement in lower culture volumes, i.e., 500 *μ*l to 300 *μ*l (Fig. 2C). Further, as the shaking frequency was increased from 650 rpm to 850 rpm, improved mixing was observed with enhanced growth (OD_600_ 43 to 50) in 600 *μ*l to 300 *μ*l culture volumes (Fig. 2A) without cells settlement in 300 *μ*l to 700 *μ*l (Fig. 2D). An additional increase in shaking frequency, i.e., 1000 rpm resulting in keeping the cells in suspension for all the culture volumes tested (Fig. 2A,E). The high cell density of OD_600_ 43 to 57 or 11 to 15 g/L DCW was achieved in all the tested culture volumes (1000 *μ*l to 300 *μ*l) at 1000 rpm in U-bottom 96-DWP. Subsequently, V-bottom 96-DWP was also tested at 3 mm, 1000 rpm shaking condition and OD_600_ value (i.e., 44 to 60 or 11.25 to 15.35 g/L DCW) was found to be similar to that of U-bottom 96-DWP (compare Figs. 2 and S2). The attained growth is equivalent to cell densities of *P. pastoris* generally achieved in adequately aerated shake flask culture^3^. Moreover, there was no spillage of culture on the cover tape or in the adjacent wells filled with sterile media while shaking up to 1000 rpm.

**Figure 3:**
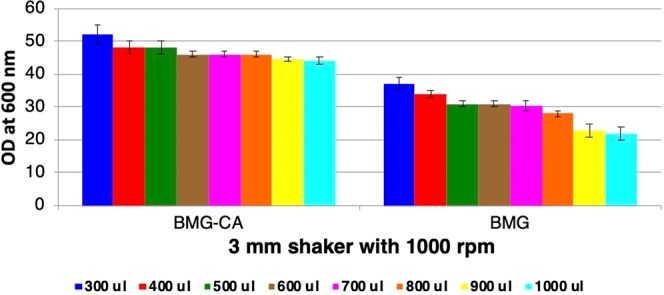
Effect of media composition on the cell density of *P. pastoris* culture in 96-DWP. Cultures in different volumes (300 µl to 1000 µl) per well of 96-square DWP (U-bottom) were grown in minimal media with (BMG-CA) and without casamino acids (BMG), in 3 mm throw diameter shaker at 1000 rpm. Aliquots were withdrawn after 72 h of cultivation and OD were measured at 600 nm. OD_600_ of the cultures are plotted on the y-axis. Different colour bars represent different culture volume per well. Each data point represents the average of 2 independent experiments.

As the initial optimization experiments were performed in YPD complex media, next, we evaluated transferability of results in buffered minimal media (BMG). Cultures were grown in U-bottom 96-DWP with shaking at 1000 rpm on 3 mm throw diameter shaker. Biomass level was found to be lower in BMG compared to complex YPD media, for example, approximately 1.6-fold lower at 600 *μ*l of culture volume (compare Figs. 2 and 3). Upon supplementation of minimal media with casamino acids (CA), an approximately 1.5-fold increase in the cell growth was observed compared to minimal media without supplementation (Fig. 3). Next, we determined the cell viability of cultures grown in different media, i.e., YPD, BMG, and BMG-CA. For this purpose, cultures from 3 mm throw diameter shaker (1000 rpm) were used. It was observed that the cell viability was >97% in both YPD and BMG-CA media, contrary to BMG with cell viability of ~84% (Fig. S3). This data shows that CA supplementation improves the performance of minimal media, bridging the cell growth gap between minimal and complex YPD media both in terms of cell growth and viability.

### Interference of media components on the binding of the secreted recombinant protein on the polystyrene surface

**Figure 4:**
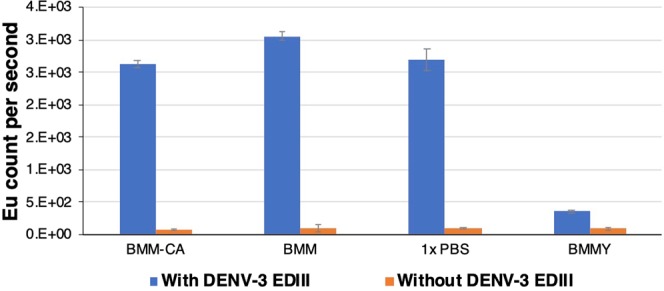
Evaluating interference of media components on the binding of recombinant protein on the polystyrene wells. Purified DENV-3 EDIII was spiked in different induction media (BMM-CA, BMM and BMMY) and PBS to 10 *μ*g/ml. The spiked media was further diluted 1:100 in 50 mM carb-bicarb buffer (pH 9.6) and 100 µl was coated on maxisorp polystyrene plate wells (effective amount 10 ng protein per well). Anti-6×-His mAb and europium labeled goat anti-mouse IgG were used as primary and secondary antibody, respectively. Error bar represents the standard deviation calculated from 4 technical replicates. Y-axis represents the Europium counts.

Possibility to directly immobilize the secreted protein product on polystyrene microtiter wells would be highly useful for the rapid screening of expression levels. To identify any interference of media components for the binding of secreted recombinant protein on polystyrene surface of immunoassay plate, purified DENV-3 EDIII was spiked in different induction media (BMM-CA, BMM, and BMMY), and PBS, and further diluted in the carbonate-bicarbonate buffer for coating on maxisorp polystyrene wells. The coating efficiency was determined using anti-his antibody in conjunction with europium labeled anti-mouse IgG. The result shows that complex BMMY media, that is often used as induction media, interfere in the binding of recombinant protein on the polystyrene surface (Fig. 4). CA supplementation of minimal media has no adverse effect on protein binding on the polystyrene surface, and the binding efficiency found to be similar to BMM media and PBS.

Based on growth and viability results in conjunction with ease of direct coating of diluted culture supernatant on the polystyrene wells, CA supplemented BMG media was chosen for further studies.

### Optimized expression conditions in 96-DWP

We determined the culture duration required to achieve a uniform growth in each well for the case where expression screening would be performed using recombinant *P. pastoris* colonies. In this experiment, we inoculated *P. pastoris* colonies of different sizes into 600 µl of media per well of the 96-DWP (U-bottom) and incubated for 96 h in a 3 mm throw diameter shaker at 1000 rpm. Aliquots were withdrawn at 24 h interval, and OD_600_ was measured. The cell density in each of the culture of different initial inoculum (colony size) reached a similar level at 48 h (Fig. S4). This suggests that 48 h is sufficient to obtain uniform maximal growth in each well of the plate, thereby would minimize the variation between individual colonies (different biomass) picked from the agar plate during the screening.

Further, we assessed the possibility of variation in the expression level of recombinant protein by identical clone in different wells of 96-DWP. For this purpose, a pre-existing *P. pastoris* clone for secretory expression of DENV-3 EDIII under the control of inducible AOX1 promoter was used. Biomass generated in BMG-CA media was induced and culture supernatant was harvested by centrifugation. No significant well-to-well variation in the secretion level was observed (Fig. S5).

### Application of the microscale cultivation method for expression screening of recombinant *P. pastoris* clones

**Figure 5:**
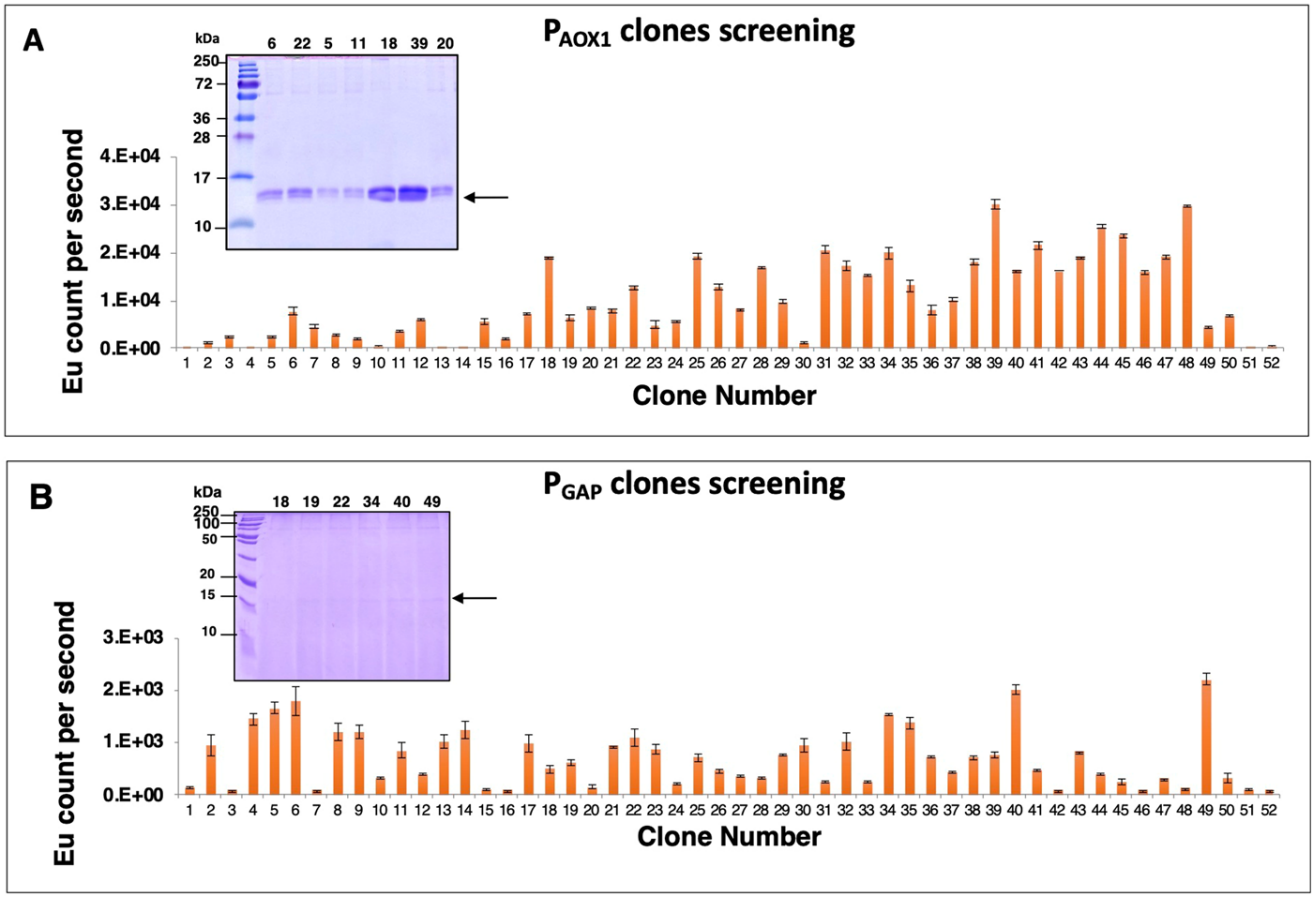
Screening of recombinant *P. pastoris* clones for the secretory expression of EDIII of DENV-1. *P. pastoris* clones were cultivated and induced according to the established microscale cultivation procedure in 96-DWP. Levels of EDIII in culture supernatants was estimated by immunoassay. Anti-6×-His mAb and europium labeled goat anti-mouse IgG was used as primary and secondary antibody, respectively. In both the panels, x-axis and y-axis represent the clone number and corresponding EDIII levels in terms of europium counts, respectively. Error bar indicates the standard deviation of 2 technical replicates. Panel A represents the clones expressing EDIII under the control of inducible promoter AOX1, with methanol induction. The panel B represents the clones expressing EDIII under the control of constitutive promoter GAP. In the panels A and B, number 51 and 52 represent negative control (KM71H cells transformed with pPICZαA). Insets depict the SDS PAGE image of secreted EDIII protein (~14 kDa) in the culture medium by different clones. The arrow on the right denote the position of the ~14 kDa EDIII protein.

To test the applicability of the established microscale cultivation procedure, we generated two constructs of dengue virus serotype-1 envelope domain III (DENV-1 EDIII) to be expressed under the control of different promoters (inducible and constitutive). *P. pastoris* codon optimized, DENV-1 EDIII gene was cloned in frame with the α-mating factor secretion sequence under the control of inducible AOX1 promoter or constitutive GAP promoter. Linearized plasmids were electroporated into *P. pastoris*, and transformants were selected on zeocin. Further, to identify the best secretory clone, colonies were grown in U-bottom 96-DWP in 3 mm throw diameter shaker with 1000 rpm, and EDIII was expressed as described in methods section. Culture supernatants from DWP were used to determine secretion level of EDIII by immunoassay. For most of the clones, EDIII protein was successfully secreted. PAOX1 based clones showed an overall higher level of EDIII secretion in comparison to the constitutive PGAP based clones (Fig. 5). The culture supernatants of few PAOX1 and PGAP-based clones were also analyzed by SDS-PAGE, and the same conclusion was drawn as observed with the immunoassay (Fig. 5). Among the screened DENV-1 EDIII clones of AOX1 promoter-based construct, a low expressor (clone #5) and a high expressor (clone #39) clone was selected for copy number determination using qPCR^18^. The quantification of integrated expression cassettes showed that low expressor clone #5 has 1 copy of the expression cassette while high expressor clone #39 has 8 copies.

**Figure 6:**
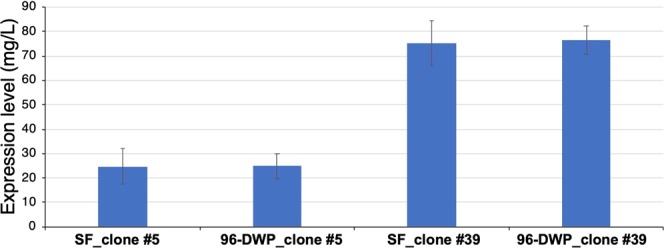
Expression of DENV-1 EDIII in shake flask and 96-DWP. Expression was performed in parallel of 2 selected clones (clone #5, copy 1 and clone #39, copy 8) in shake flask (SF) and square 96-DWP with U-bottom, in BMM-CA at 20 °C for a duration of 48 h. Cells were harvested, and culture supernatants were assayed for EDIII secretion using immunoassay. Level of DENV-1 EDIII in the culture supernatant was determined from the standard curve generated using purified DENV-1 EDIII as reference. X-axis represents the clone and cultivation details. Y-axis represents EDIII expression level. Clone #5 and clone #39 contain 1 and 8 gene copies, respectively. SF and 96-DWP means shake flask and 96-deep well plate, respectively.

These two clones (clone #5 and clone 39) were further used for expression in shake flask as well as in 96-DWP. We found comparable yields of secreted EDIII in both shake flask and 96-DWP setup (Figs. 6 and S6).

## Discussion

Despite the fact that *P. pastoris* is an important recombinant protein production host, only a few studies for microscale cultivation of this host have been reported so far^8–11^. Moreover, in most of these studies, there is no comprehensive information on the degree to which these methods were successful. Only a few reports like Weis *et al*., 2004 and Qin *et al*. 2011 have reported cell density, OD_600_ 10 to 12 and DCW 3.3 g/L, respectively, that was achieved^9,10^. These values are 5–6 times lower than the cell density often achieved in shake flask (OD_600_ 50 to 60)^3^. Large differences in the cell growth in microscale and scale-up may result in a false or suboptimal selection of clone or conditions in the screening process, and thereby, inconsistent performance of the selected clone or conditions upon scale-up^20^. Moreover, enhanced cell density at micro-well cultivation setup is required for high throughput production and purification of recombinant proteins or variants from *P. pastoris* for diverse applications.

The major problem with microbial cultivation in the DWP has been the inadequate oxygen supply due to poor mixing^13,21^. In this context, it becomes essential to perform a comprehensive evaluation of multiple parameters for optimized mixing in micro-well. Therefore, the current work tries to address this need. We, here, first time evaluated the impact of multiple factors like shaking throw diameter, shaking frequency, plate geometry, culture volume per well and media composition on the growth and viability of *P. pastoris* cells. The growth of *P. pastoris* was compared in 96-square DWP of different bottom geometry (V- and U-shaped), wells filled with culture volumes from 300 *μ*l to 1000 *μ*l and incubated under different shaking conditions (300 rpm in 25 mm and 50 mm throw diameter shaker, and 400 rpm in 25 mm throw diameter shaker). We could not test more than 400 rpm with 25 mm throw diameter shaker and more than 300 rpm shaking frequency with 50 mm throw diameter shaker because of the strong vibration in the shaker.

The results demonstrate an increase in *P. pastoris* growth of about 5.3-fold in 300 *μ*l to 600 *μ*l and about 3-fold in 700 *μ*l to 1000 *μ*l in V-bottom, and about 6.3-fold in 300 *μ*l to 700 *μ*l and about 4.1-fold in 800 *μ*l to 1000 *μ*l in U-bottom 96-DWP on 50 mm throw shaker in comparison to 25 mm throw diameter shaker with 300 rpm (Fig. 1). Similar observations have been reported by Duetz *et al*.^21,22^. It has been speculated that the centrifugal force generated by 25 mm amplitude orbital shaking at 300 rpm is not sufficient to induce the enough motion and consequently, most of the liquid does not circulate in phase with the orbital motion of the shaking platform^22^. This results in a lower oxygen mixing and lower cell growth. While 50 mm amplitude can lead to turbulent flow causing an increase in oxygen mixing in the culture and thereby results in higher cell growth^22^.

Another approach to generate turbulent flow pattern in the wells is to increase the shaking frequency without change in throw diameter^13,23^. When shaking frequency of 25 mm throw diameter shaker was increased from 300 rpm to 400 rpm (maximum shaking possible), there was increase in the cell growth about 6.9-fold increase in 300μl to 600 μl and about 4.5-fold increase in 700 μl to 1000 μl of culture volumes in V-bottom wells, whereas, about 7.7-fold increase in 300 μl to 600 μl and about 7-fold increase in 700 μl to 1000 μl of culture volumes in U-bottom wells. This comparison shows that shaking in 25 mm throw diameter shaker at 400 rpm result in higher growth even when compared to shaking at 50 mm throw shaker at 300 rpm.

Besides the centrifugal force induced by shaker, the geometry of the 96-DWP also plays a crucial role in keeping the cells in suspension. Since, the corners of the square DWP act as a baffle and contribute to high oxygen supply by causing turbulent flow pattern in comparison to round shaped wells^13,21^, we have only used square DWP in this study. The combination of the square well with the flat bottom is considered to be ideal in providing the adequate oxygen supply^10,22^. However, we have not used the flat-bottom plate because of the problem of dislodging of the cell pellet after centrifugation, causing difficulty in separating the clarified supernatant from the pellet for subsequent experiments. We have instead performed a comparative study between square well with U- and V-bottom.

Despite the turbulent shaking pattern generated by 50 mm throw diameter shaker at 300 rpm and 25 mm throw diameter shaker at 400 rpm, higher cell settlement was observed in V-bottom in comparison to U-bottom 96-DWP (Fig. 1). For example, cells were settled in all the tested culture volumes in V-bottom, on 50 mm throw shaking, compared to U-bottom wells. Similarly, in 25 mm throw shaking at 400 rpm, higher cell settlement was observed in V-bottom wells compared to U-bottom wells (Fig. 1). Overall higher growth (OD_600_) was also achieved in U-bottom wells. Cell settlement and lower growth in the V-bottom well may be because of the poor mixing in the bottom part of the well that is without the baffle and much deeper and narrower than U-bottom plate. Square DWPs with U-bottom provides growth benefit while keeping the cells in suspension and easy separation of cell pellet and supernatant after centrifugation. U-bottom DWP provides the advantage of both the flat bottom plate (adequate mixing) and V-bottom plate (easy separation of cell pellet and supernatant after centrifugation). Despite the adequate growth in 25 mm throw diameter shaker with 400 rpm, higher culture volumes (>800 µl) resulted in lower cell growth and settlement of cells even with U-bottom wells (Fig. 1).

Next, we tested culture mixing on 3 mm throw diameter shaker with different shaking frequencies (500, 650, 850, 1000 rpm) with U-bottom plate, to enhance the cell growth. As can be seen from Fig. 2 that there was an increase in cell growth and reduction in cell settlement with an increase in shaking frequency with 3 mm throw shaker; the optimal results were obtained with 1000 rpm resulting in the highest growth without cells settlement even at 1000 µl/well volume. When 1000 rpm shaking frequency was used for V-bottom well plate, similar growth was observed as compared to U-bottom plate (Fig. S2). This suggests that 1000 rpm in 3 mm throw diameter shaker is sufficient to keep the cells in suspension even for the V-bottom plate.

Having selected 3 mm throw diameter shaker with 1000 rpm and U-bottom 96-DWP, next, we investigated the impact of media composition on the cell growth and viability. The cell density of OD_600_ > 50 (Figs. 2 and 3) and viability of >97% were obtained in complex YPD media as well as in CA supplemented buffered minimal media (BMG-CA) (Fig. S3). Contrary to this, poor growth (Fig. 3) and cell viability (Fig. S3) were observed in buffered minimal media (BMG) without CA supplementation in DWP. This indicates that the supplementation of BMG with CA, that is a ready-made source of amino acids and nitrogen, enhanced the cell growth and bridge the gap observed between non-supplemented BMG and complex YPD media. Earlier we have reported that the presence of CA in culture media doesn’t interfere in the downstream protein purification methods^3^. In the present study, we have also found that CA does not interfere (or compete) in the binding of recombinant protein on the polystyrene wells unlike yeast extract and peptones in complex media (Fig. 4). The Bacto casamino acids, used by us, primarily contains free amino acids (about 90%) and some very small peptides of less than 5 amino acids^24^, whereas complex media components like peptones, that compete for binding on polystyrene surface, is mixture of majorly (about 80%) long peptides and very few free amino acids (about < 14%)^25^. The non-binding property of CA to the polystyrene well is particularly advantageous for the screening of clones for expression level as the culture supernatant can directly be coated on the polystyrene wells for the immunoassay without the need for specific capture surface, like wells coated with protein-specific antibodies or Ni-NTA (for the capture of His-tagged protein). The successful adaptation of microscale cultivation method for minimal media allows this method to be used for high throughput screening as well as high throughput protein production and purification without the problem of co-purification of components from complex media.

This represents the first report where such a high cell density (OD_600_ 50 or DCW 13 g/L) of *P. pastoris* was attained in buffered minimal media in 96-DWP containing culture volumes up to 1000 *μ*l per well. The obtained biomass yield is about 5-fold higher than the previously reported biomass in 96-DWP^10^ and 48-well plate^9^ in minimal media.

There was no noteworthy well-to-well variation in growth and viability indicating that centrifugal force is identical in each well providing similar oxygen supply throughout the plate (corners as well as the middle) and therefore cell growth shows negligible variation.

As the large incubator shaker with 3 mm throw diameter is commonly not available in the research laboratories, we tested the transferability of the results obtained with *P. pastoris* culture with easy to use compact orbital plate shakers of 3 mm throw diameter such as MixMate (Eppendorf, GmBH, model no. 5353000510) that can hold one plate and orbit P4 digital shaker (Labnet International. Inc, NJ, model no. S2020-P4-B) that can hold up to 4 plates by placing these plate shakers inside a temperature-controlled incubator. We found similar cell growth at shaking frequency of 1000 rpm on MixMate shaker and 1100 rpm on P4 shaker without any spillage up to 1000 *μ*l of culture volume per well (data not shown). This suggests that simple compact plate shaker can also be used for the enhanced cell density cultivation of *P. pastoris* in DWP. Moreover, in case of low culture volume (upto 800 µl), routine 25 mm throw diameter shaker at 400 rpm is sufficient to achieve enhanced cell density (Fig. 1E,H).

Due to the differences in the colony size or operator level variation, there can be variation in the biomass amount inoculated in different wells from the agar-plate containing the transformed colonies. We determined that cell density for each of the culture of different initial inoculum (colony size) reaches a similar level at 48 h (Fig. S4). This data suggests that 48 h is sufficient to obtain uniform maximal growth in each well of the plate, thereby would minimize the variation between individual colonies (different biomass) picked from the agar plate during screening.

We also assessed the applicability of the developed microscale cultivation method for the screening of recombinant *P. pastoris* transformants intended to secrete DENV-1 EDIII under the control of inducible AOX1 or constitutive GAP promoter. Our results show that the developed microscale cultivation method is suitable for both the inducible and constitutive promoter-based *P. pastoris* clones (Fig. 5). Moreover, we have observed higher secretion level from clones having EDIII gene under the control of inducible AOX1 promoter compared to constitutive GAP promoter. This observation is not unique as often the AOX1 promoter performs better than GAP promoter in *P. pastoris*^26–28^. Furthermore, in the enhanced cell density microscale cultivation setup, different EDIII secretion levels among different clones were observed (Fig. 5). This observed variability is often because of differences in the copy number of expression cassette integrated into the *P. pastoris* genome^29^. We have determined the copy number of a low expressor (clone#5) and a high expressor (clone #39) AOX1 promoter-based clone, which found to contain 1 and 8 copies, respectively. This emphasize the necessity for an efficient and reliable microscale screening method for *P. pastoris* to screen and identify the so-called jackpot clone.

We have observed similar expression level in shake flask and DWP, when the low expressor (clone #5, copy 1) or the high expressor (clone #39, copy 8) clone of DENV-1 EDIII under the control of AOX1 promoter was cultivated (Fig. 6). This shows that the devised enhanced cell density microscale cultivation and expression-screening strategy is an efficient method for the identification of recombinant clones. Moreover, given the significance of scalability of cultivation results from microscale to laboratory scale-up flask, the method described herein will be of immense value to laboratories that are not equipped with microbioreactor facility for performing parallel screening of clones or culture conditions. Our optimized method can also be useful for screening of a library of recombinant antibody or enzyme mutants to identify leads with improved affinity and specificity or activity. The developed method is not limited to the screening of clones but can also be used for the optimization of culture conditions, *e.g*. screening of different media components and additives often required for the stability of the secretory protein^3^. Moreover, the developed microscale cultivation method can be adapted for other cell suspension cultures.

## Supplementary information

Supplementary information.

## References

[1] Damasceno LM, Huang CJ, Batt CA (2012). Protein secretion in *Pichia pastoris* and advances in protein production. Appl Microbiol Biotechnol.

[2] Macauley-Patrick S, Fazenda ML, McNeil B, Harvey LM (2005). Heterologous protein production using the *Pichia pastoris* expression system. Yeast.

[3] Kaushik N (2016). Casamino acids facilitate the secretion of recombinant dengue virus serotype-3 envelope domain III in *Pichia pastoris*. BMC Biotechnol.

[4] Schwarzhans J-P, Luttermann T, Geier M, Kalinowski J, Friehs K (2017). Towards systems metabolic engineering in *Pichia pastoris*. Biotechnology Advances.

[5] Bruhlmann D (2017). Parallel experimental design and multivariate analysis provides efficient screening of cell culture media supplements to improve biosimilar product quality. Biotechnol Bioeng.

[6] Shah KA (2015). Automated pipeline for rapid production and screening of HIV‐specific monoclonal antibodies using *pichia pastoris*. Biotechnology and bioengineering.

[7] Koskela, E. V., de Ruijter, J. C. & Frey, A. D. Following nature’s roadmap: folding factors from plasma cells led to improvements in antibody secretion in *S. cerevisiae*. *Biotechnology journal* **12** (2017). 10.1002/biot.201600631 28429845

[8] Boettner M, Prinz B, Holz C, Stahl U, Lang C (2002). High-throughput screening for expression of heterologous proteins in the yeast *Pichia pastoris*. J Biotechnol.

[9] Qin X (2011). Reliable high‐throughput approach for screening of engineered constitutive promoters in the yeast *Pichia pastoris*. Letters in applied microbiology.

[10] Weis R (2004). Reliable high-throughput screening with *Pichia pastoris* by limiting yeast cell death phenomena. FEMS yeast research.

[11] Barnard GC (2010). High-throughput screening and selection of yeast cell lines expressing monoclonal antibodies. J Ind Microbiol Biotechnol.

[12] Zimmermann HF, Anderlei T, Büchs J, Binder M (2006). Oxygen limitation is a pitfall during screening for industrial strains. Applied microbiology and biotechnology.

[13] Klöckner W, Büchs J (2012). Advances in shaking technologies. Trends in biotechnology.

[14] Micheletti M, Lye GJ (2006). Microscale bioprocess optimisation. Current opinion in biotechnology.

[15] Chávez JH, Silva JR, Amarilla AA, Figueiredo LTM (2010). Domain III peptides from flavivirus envelope protein are useful antigens for serologic diagnosis and targets for immunization. Biologicals.

[16] Lin-Cereghino, J. *et al*. Condensed protocol for competent cell preparation and transformation of the methylotrophic yeast *Pichia pastoris*. *Biotechniques* **38**, 44, 46, 48 (2005). 10.2144/05381BM04 PMC2504082 15679083

[17] De Schutter K (2009). Genome sequence of the recombinant protein production host *Pichia pastoris*. Nature biotechnology.

[18] Abad S (2010). Real‐time PCR‐based determination of gene copy numbers in *Pichia pastoris*. *Biotechnology*. Journal: Healthcare Nutrition Technology.

[19] Batra G (2010). Optimization of conditions for secretion of dengue virus type 2 envelope domain III using *Pichia pastoris*. Journal of bioscience and bioengineering.

[20] Looser, V. *et al*. Cultivation strategies to enhance productivity of *Pichia pastoris*: A review. *Biotechnol Adv*, 10.1016/j.biotechadv.2015.05.008 (2015). 10.1016/j.biotechadv.2015.05.008 26027890

[21] Duetz WA (2000). Methods for intense aeration, growth, storage, and replication of bacterial strains in microtiter plates. Applied and environmental microbiology.

[22] Duetz WA, Witholt B (2001). Effectiveness of orbital shaking for the aeration of suspended bacterial cultures in square-deepwell microtiter plates. Biochemical Engineering Journal.

[23] Duetz WA (2007). Microtiter plates as mini-bioreactors: miniaturization of fermentation methods. Trends in microbiology.

[24] BD. *Bacto Casamino Acids*, http://www.bdbiosciences.com/ds/ab/others/Casamino_Acids.pdf.

[25] BD. *Bacto Peptone*, http://www.bdbiosciences.com/ds/ab/others/Bacto_Peptone.

[26] Sears IB, O’Connor J, Rossanese OW, Glick BS (1998). A versatile set of vectors for constitutive and regulated gene expression in *Pichia pastoris*. Yeast.

[27] Vassileva A, Chugh DA, Swaminathan S, Khanna N (2001). Expression of hepatitis B surface antigen in the methylotrophic yeast *Pichia pastoris* using the GAP promoter. Journal of biotechnology.

[28] Boer H, Teeri TT, Koivula A (2000). Characterization of Trichoderma reesei cellobiohydrolase Cel7A secreted from *Pichia pastoris* using two different promoters. Biotechnol Bioeng.

[29] Ahmad M, Hirz M, Pichler H, Schwab H (2014). Protein expression in *Pichia pastoris*: recent achievements and perspectives for heterologous protein production. Appl Microbiol Biotechnol.

