# Supplementary material for: Enhanced cell density cultivation and rapid expression-screening of recombinant Pichia pastoris clones in microscale
Source: Sci Rep. 2020 May 4;10:7458. doi: 10.1038/s41598-020-63995-5 (PMC7198582; doi:10.1038/s41598-020-63995-5)
Supplement: Supplementary file 1 — Supplementary information. [file 41598_2020_63995_MOESM1_ESM.pdf]

# Enhanced cell density cultivation and rapid expression-screening of recombinant *Pichia pastoris* clones in microscale

Neha Kaushik, Urpo Lamminmäki, Navin Khanna, and Gaurav Batra[1*]

## Supplementary Information

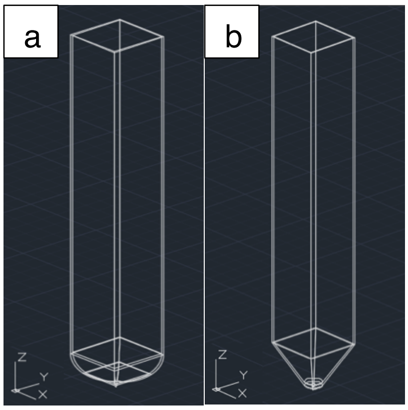


**Figure S1. Three-dimensional view of deep well.** It depicts square shaped well with (a) U-shaped and (b) V-shaped bottom geometry.

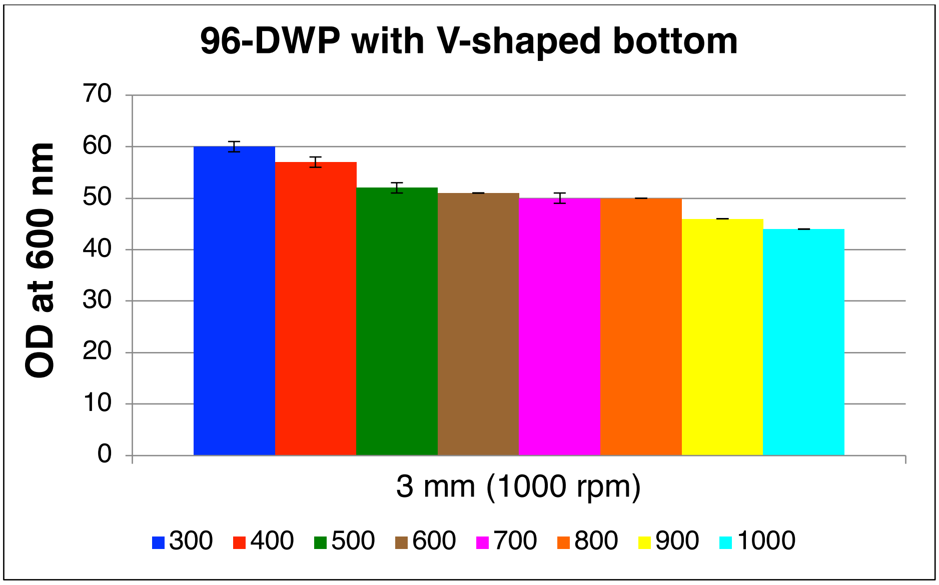


**Figure S2. Shaking of *P. pastoris* culture in square well with V-shaped bottom 96-DWP on 3 mm throw diameter shaker with 1000 rpm shaking frequency.** Cells were grown in different culture volumes (300µl to 1000 µl per well) in YPD media in square 96-DWP with V-bottom. Aliquots were withdrawn after 72 h of cultivation and OD were measured at 600 nm. $OD_{600}$ of the cultures is plotted on the y-axis. Different colour bars represent different culture volume per well. Each data point represents the average of the 3 independent experiments.

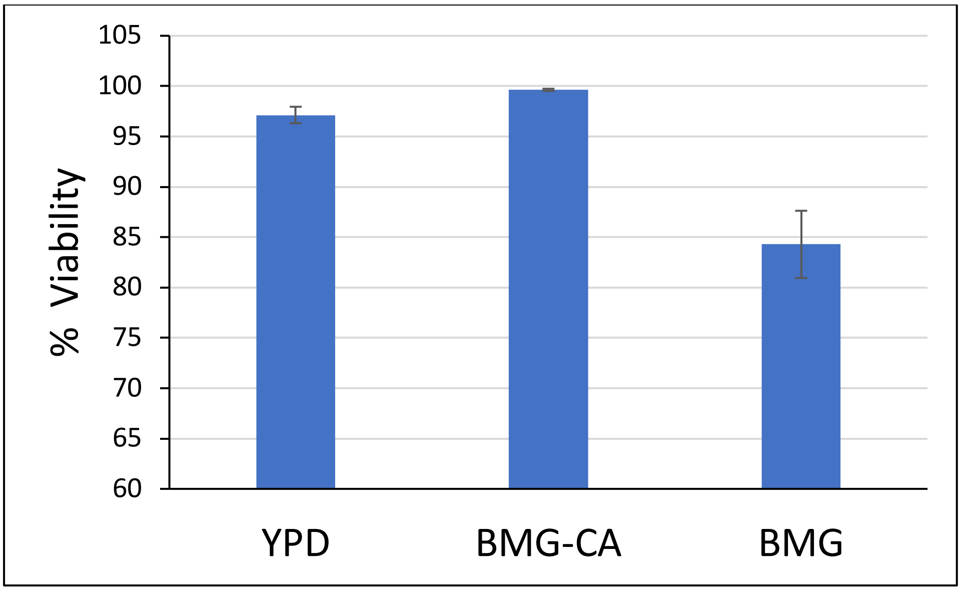


**Figure S3. Comparison of cell viability of cultures grown in different growth media in 96-DWP.** Cultivation was done in square 96-DWP with U-bottom in different media (YPD, BMG, and BMG-CA) for 72 h. The proportion of dead and live cells were determined by propidium iodide and syto9 dyes using flow cytometry. Results shown in percentage represent the average of 3 independent experiments. Each experiment was having 2 technical replicates.

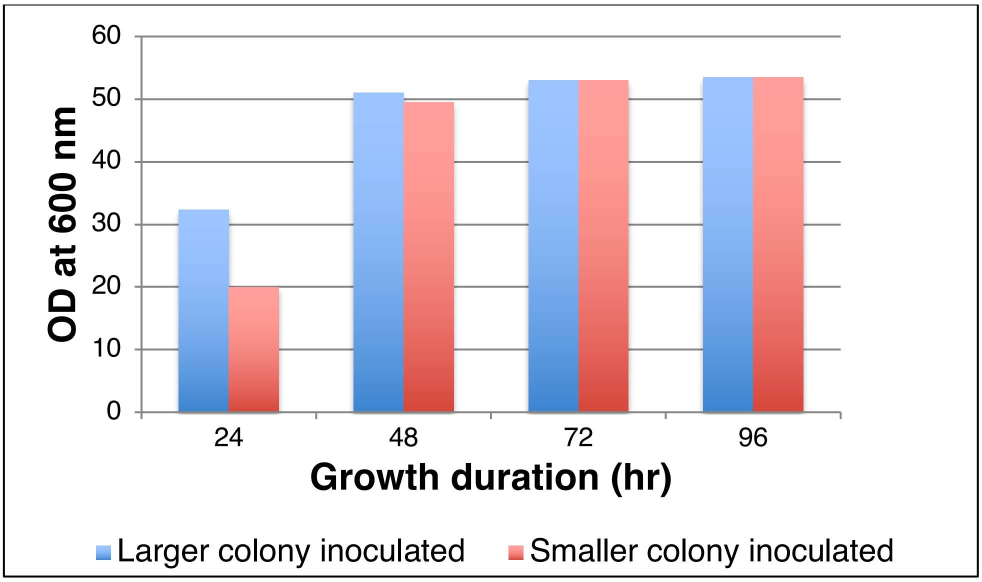


**Figure S4. A time course of growth of *P. pastoris* KM71H in YPD medium.** Small and large size colony of *P. pastoris* KM71H from YPD agar plate was inoculated into 600 µl of YPD medium in 96-DWP. Plate was incubated on 3 mm throw diameter shaker at 1000 rpm for 96 h at 30°C. Culture aliquots were withdrawn at interval of every 24 h and optical density was measured at 600 nm.

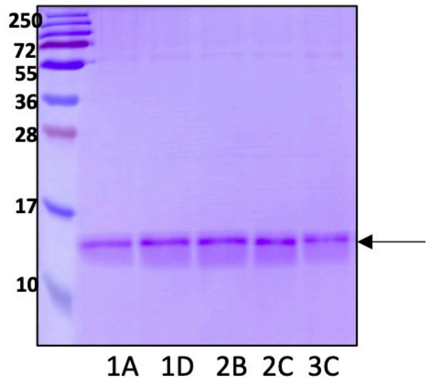


**Figure S5. Coomassie-stained SDS-PAGE showing the presence of secreted EDIII of DENV-3 in culture supernatant from different wells of 96-DWP.** Identical clone of recombinant *P. pastoris* harboring DENV-3 EDIII expression cassette (where, EDIIII gene was under the control of inducible AOX1 promoter). Culture was harvested at 48 h post induction and levels of secreted EDIII in different wells (Well no. 1A, 1D, 2B, 2C and 3C) of 96-DWP were determined. The arrow at the right indicates the position of EDIII.

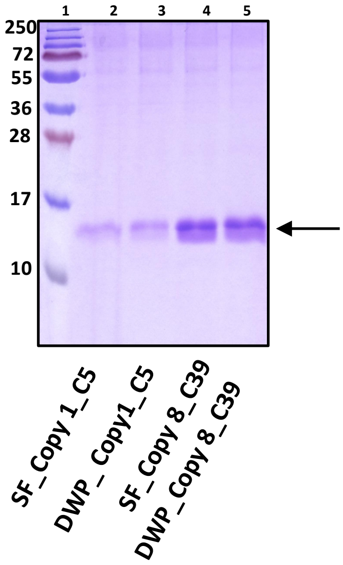


**Figure S6. Comparison of low and high expressor *P. pastoris* clone of DENV-1 EDIII.** *P. pastoris* C5 (clone#5, harbouring 1 gene copy) and C39 (clone#39, harbouring 8 gene copies) were expressed under the control of inducible AOX1 promoter in shake flask (SF) and 96-DWP. Coomassie-stained SDS-PAGE is showing the presence of secreted EDIII in culture supernatant. The arrow at the right indicates the position of EDIII.
